# Padua Prediction Score and Hospital-Acquired Proximal and Isolated Distal Deep Vein Thrombosis in Symptomatic Patients

**DOI:** 10.3390/hematolrep16040055

**Published:** 2024-09-25

**Authors:** Michelangelo Sartori, Miriam Fiocca, Mario Soldati, Laura Borgese, Elisabetta Favaretto, Benilde Cosmi

**Affiliations:** 1 Angiology and Blood Coagulation Unit, IRCCS Azienda Ospedaliero-Universitaria di Bologna, 40138 Bologna, Italy; mario.soldati@aosp.bo.it (M.S.); laura.borgese@aosp.bo.it (L.B.); elisabetta.favaretto@aosp.bo.it (E.F.); 2 Department of Medical and Surgical Sciences, University of Bologna, IRCCS Azienda Ospedaliero-Universitaria di Bologna, 40138 Bologna, Italy; miriam.fiocca@studio.unibo.it; 3 Angiology and Blood Coagulation Unit, Department of Medical and Surgical Sciences, University of Bologna, 40138 Bologna, Italy; benilde.cosmi@unibo.it

**Keywords:** deep vein thrombosis, diagnosis, inpatients, isolated distal deep vein thrombosis, calf deep vein thrombosis, Padua prediction score

## Abstract

Background: Hospital-acquired deep vein thrombosis (DVT) is an important cause of morbidity and mortality. Objectives: The purpose of this study was to evaluate the prevalence of proximal lower limb DVT and isolated distal DVT (IDDVT) and their relationship to the Padua Prediction Score (PPS) in acutely ill, hospitalized patients. Methods: In a single-center cross-sectional study, all inpatients from medical departments with suspected lower-extremity DVT were evaluated with whole-leg ultrasonography during 183 days from 2016 to 2017. Results: Among the 505 inpatients (age 78.0 ± 13.3, females 59.2%) from medical departments, 204 (40.2%) had PPS ≥ 4, but only 54.4% of them underwent pharmacological thrombo-prophylaxis. Whole-leg ultrasonography detected 47 proximal DVTs (9.3%) and 65 IDDVTs (12.8%). Proximal DVT prevalence was higher in patients with high PPS vs. those with low PPS (12.7% vs. 7.0% *p* = 0.029, respectively), whereas IDDVT prevalence was similar in patients with high and low PPS (14.7% vs. 11.6% *p* = 0.311, respectively). The area under the receiver operating curve (AUC) for the PPS was 0.62 ± 0.03 for all DVTs, 0.64 ± 0.04 for proximal DVTs, and 0.58 ± 0.04 for IDDVTs. Conclusions: In hospitalized patients, IDDVT had similar prevalence regardless of PPS risk stratification. Adherence to thrombo-prophylaxis in patients was still far from optimal.

## 1. Introduction

Hospital-related venous thromboembolism (VTE) is a major cause of long-term morbidity, functional disability, and mortality [1]. Recently, we showed that isolated distal or calf vein thrombosis (IDDVT) is a frequent finding in hospitalized patients [2]. Although IDDVT is a more benign condition than proximal DVT, it may extend to proximal veins or may lead to pulmonary embolism (PE) if left untreated [3].

Past guidelines recommend anticoagulant thrombo-prophylaxis with low-molecular-weight heparin for acutely ill, hospitalized patients at increased risk of thrombosis [4] and suggest a risk assessment model based on the Padua Prediction Score (PPS) for baseline low- and high-risk stratification [4]. In the original study of Barbar et al., patients with a PPS > 4 experienced 30 times more VTE complications compared with the low-risk group [5]. However, the PPS was empirically created on experts’ opinion and literature reviews and was validated in a population that included patients receiving thrombo-prophylaxis [5]. In a retrospective analysis of patients enrolled in the PREVENU trial, the PPS’s performance was not superior to evaluating the patient’s age alone in VTE risk assessment [6]. Nevertheless, the PPS is applied extensively in medical departments in Emilia Romagna [7], and its use is encouraged by the latest guidelines [8].

Since no data are available on the PPS’s ability to accurately identify inpatients at risk of IDDVT, the purpose of this study was to evaluate the prevalence of lower limb DVT in different risk stratification groups based on the PPS in hospitalized patients from medical wards.

## 2. Materials and Methods

### 2.1. Study Setting

This was an ancillary analysis of an observational study performed in a Tertiary Healthcare Academic Hospital (IRCCS Azienda Ospedaliero-Universitaria di Bologna) from 1 October 2016 to 31 March 2017 [2]. This study sought to describe the prevalence of symptomatic lower limb DVT in hospitalized patients from medical wards and the accuracy of the PPS in patients with suspected lower limb DVT. This study was approved by the local Ethics Committee. Written informed consent was obtained from all the patients. All the procedures performed in this study involving human participants were conducted in accordance with the ethical standards of the institutional and/or national research committee and with the 1964 Helsinki declaration and its later amendments or comparable ethical standards.

### 2.2. Study Population

All of the patients hospitalized in medical wards for acute illness who were suspected of DVT after admissionwere eligible for this study. Patients were excluded if they had DVT symptoms before hospital admission, in cases of hospitalization for DVT and/or PE or a diagnosis of DVT or PE in the previous 12 months, if younger than 18 years, pregnant or puerperium, and if they had surgery or leg fracture or used a plaster cast within 3 months before admission, whereas patients with minor trauma of the symptomatic leg were included. Moreover, we excluded patients who were already receiving anticoagulants as vitamin K antagonists or direct oral anticoagulants before admission. All consecutive eligible patients were enrolled during business days. Acute illness was defined as acute congestive heart failure, acute respiratory disease, infection, acute exacerbation phase of an inflammatory disease, stroke, critical limb ischemia, acute pancreatitis, peptic ulcer disease, hepatic decompensation, and diabetic decompensation. The patients enrolled in this study were considered to have the following: (i) acute respiratory failure if they were admitted to the medical ward for acute hypoxemia due to lung failure and (ii) acute heart failure if they were admitted to the medical ward for rapid onset or worsening of symptoms and/or signs of heart failure as the first occurrence or as a consequence of acute decompensation of chronic heart failure.

At the time of admission in the medical ward, the physician in charge decided whether the patients needed prophylaxis and the type of prophylaxis according to his clinical judgment. Pharmacologic thrombo-prophylaxis was defined as the use of subcutaneous heparin calcium, 5000 U, 2 or 3 times daily, or subcutaneous enoxaparin sodium, 40 mg/day, or subcutaneous fondaparinux, 1.5/2.5 mg/day, started at admission and administered daily until whole-leg ultrasonography. Mechanical prophylaxis was defined as the use of anti-embolism stockings or intermittent pneumatic compression.

### 2.3. Study Design

**Figure 1:**
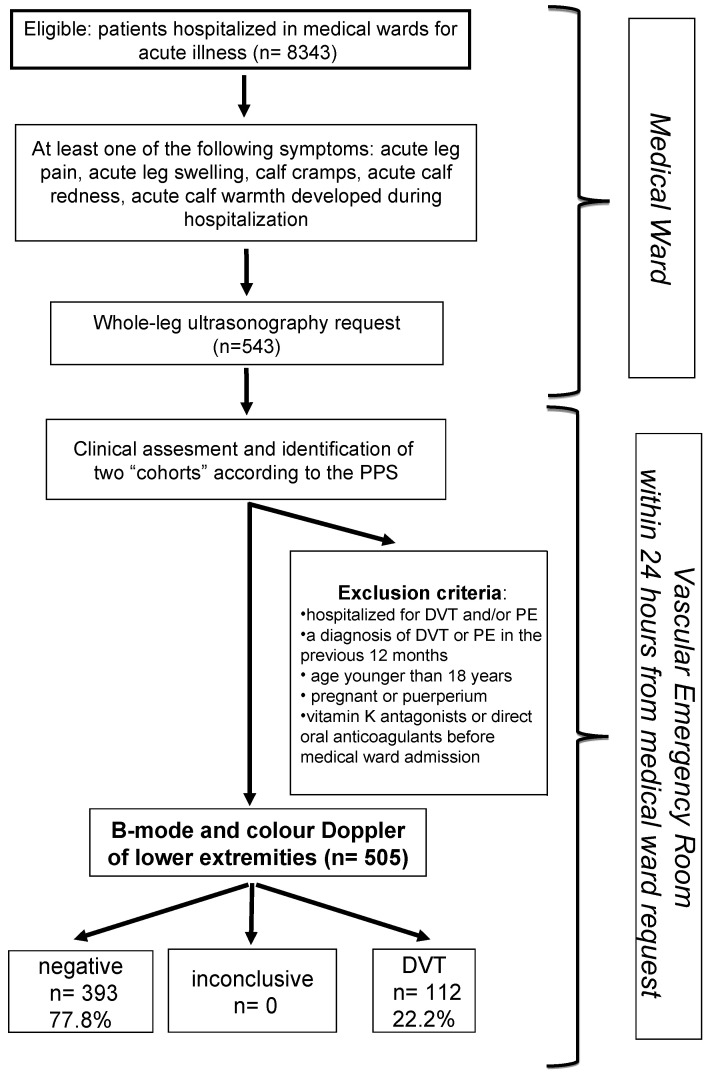
Study flow chart. DVT, lower extremity deep venous thrombosis; PE, pulmonary embolism; PPS, Padua Prediction Score.

In this observational cross-sectional study, symptomatic inpatients referred for suspected acute DVT of the lower limbs for the following acute symptoms developed after admission in the medical ward were eligible for this study: leg pain, leg swelling, calf cramps, calf redness, and calf warmth (Figure 1). During the hospital stay, in cases of suspected lower extremity DVT, the medical ward clinician ordered an ultrasound, which was performed within 24 h as whole-leg duplex ultrasonography (DUS). The patients were carried to the Vascular Emergency Service unit, which was the only reference unit in our hospital for patients with suspected DVT. In the Vascular Emergency Service unit, the patients signed their informed consent, and a separate investigator reviewed the clinical files, performed a physical examination of the patient in the supine position, and elicited personal and family histories from each patient, collecting data in standardized form and calculating the PPS. The number of days from admission (day 0) to the time when suspicion of DVT arose were calculated. Next, other physicians performed and interpreted the DUS. The diagnosis of proximal DVT and IDDVT was based on the DUS.

Heparin-induced thrombocytopenia (HIT) was suspected in all subjects receiving heparin thrombo-prophylaxis when a 50% or more drop in platelet count in comparison to the pretreatment value was observed. In cases of a platelet count drop of 50% or more, a blood sample was obtained for the determination of heparin-dependent IgG antibodies.

### 2.4. Padua Prediction Score

Among several risk assessment models for VTE in medical inpatients, past guidelines suggested the PPS [4]. The PPS includes several items. One point was added for each of the following positive findings: (a) age ≥ 70 years, (b) heart and/or respiratory failure, (c) acute myocardial infarction or ischemic stroke, (d) acute infection and/or rheumatologic disorder, (e) BMI ≥ 30, and (f) ongoing hormonal treatment; two points were added for recent trauma and/or surgery; three points were added for each of the following positive findings: (a) active cancer, (b) previous VTE, (c) reduced mobility, and (d) an already known thrombophilic condition. The overall score was obtained by the sum of each item, and a final score > 4 was considered indicative of a high risk of VTE [5].

Immobility was defined as bed rest without bathroom privileges (either because of the patient’s limitations or on a physician’s orders) for at least 3 days. The patients enrolled in this study were considered to have (i) acute respiratory failure if they were admitted to the medical ward for acute hypoxemia due to lung failure and (ii) acute heart failure if they were admitted to the medical ward for rapid onset or worsening of symptoms and/or signs of heart failure as the first occurrence or because of acute decompensation of chronic heart failure.

### 2.5. Whole-Leg Ultrasonography Investigation

In our Academic Hospital, hospitalized patients with a clinical suspicion of DVT undergo a DUS evaluation within 24 h from the request. All of the patients underwent DUS of both lower limbs by a vascular medicine physician with at least 4 years of experience performing DUS of the lower leg venous system, as previously described [9]. Briefly, the patients underwent a comprehensive real-time B-mode and color Doppler compression ultrasonography examination of both legs—first the proximal deep veins, then the calf veins. The iliac, femoral, and popliteal veins were examined first; then, in patients with normal findings, the calf veins were evaluated with the patient seated and their legs vertical. Calf veins were studied using various views, as follows: anterior–medial, posterior, and postero-lateral. The following veins were scanned in the transverse plane over their entire length: posterior tibial and fibular (axial veins), medial and lateral gastrocnemius, and soleal veins (muscular veins). DVT diagnosis was confirmed if there was a presence of endoluminal material combined with a lack of compression of the vein. Clot diameter was measured in the transverse plane during maximal compression. The results were registered on a chart according to clot location and diameter. IDDVT was defined as thrombosis of posterior tibial, fibular, medial and lateral gastrocnemius, and soleal vein, whereas thrombosis that involved the trifurcation, the popliteal vein, and/or the above venous system was defined as proximal DVT. The physician interpreting the ultrasound was blinded to the PPS.

### 2.6. Statistical Analysis

Analysis was carried out using the IBM SPSS™ software package (version 21; IBM Corp., Armonk, NY; USA). Estimated DVT prevalence in inpatients was calculated as the ratio between the number of DVTs during the study period (180 days) and the number of total hospital admissions in the medical, hematology, and oncology wards during the study period. Relationships between variables were assessed by means of Pearson correlation for continuous variables and a chi-square test for categorical variables. Student’s *t*-test was used to compare means among groups for normally distributed variables. Receiver operating characteristic (ROC) curves were determined by plotting the sensitivity vs. 1-specificity. The area under the ROC curves (AUC) for the discriminatory accuracy of the PPS was calculated. Categorical variables were expressed as frequency and percentage with 95% CI; continuous variables were expressed as mean ± SD. For the time-to-first-event analysis, cumulative endpoint curves were estimated with the Kaplan–Meier procedure, and survival curves were tested by the log-rank test. The significance level was set at <0.05.

## 3. Results

Figure 1 shows the study flowchart. The characteristics of 505 patients (age 78.0 ± 13.3 years, females 59.2%) enrolled from medical/hematology/oncology departments with suspected lower limb DVTs are summarized in Table 1. Among them, the most frequent risk factors for thrombosis were immobility (50.9%), cancer (22.3%), minor trauma involving the symptomatic leg within 1 month (14.4%), and previous VTE (13.8%). Pharmacologic thrombo-prophylaxis was administered to 282 (55.6%) patients. No HIT was diagnosed in patients receiving heparin thrombo-prophylaxis during the hospital stay. There were 301 (59.4%) patients with PPS < 4 and 204 (40.2%) with PPS ≥ 4; in two patients, the PPS was not calculated (Table 2). The percentage of subjects receiving pharmacological thrombo-prophylaxis was similar in patients with a high and a low PPS (57.2% vs. 54.2%, *p* = 0.413, respectively). DUS was performed after a mean of 5 days (median 3 days) from hospitalization, and it detected DVTs in 112 patients (22.1%); in 47 patients (9.3%), proximal DVTs were found, while in 65 (12.9%), IDDVTs were found. Among patients with IDDVT, thrombosis confined to the muscle veins was detected in 48 patients (9.5%), axial calf thrombosis was detected in 10 patients (2.0%), and both muscular and axial calf thrombosis were detected in 7 patients (1.4%). During the study period, there were 8343 hospital admissions in the medical services. The estimated prevalence of all DVTs in the hospital medical setting was 1.34 per 100 hospital admissions (95% CI: 1.11–1.61), the estimated proximal DVT incidence was 0.56 per 100 admissions (95% CI: 0.42–0.74), and the estimated IDDVT incidence was 0.78 per 100 admissions (95% CI: 0.61–0.99).

As shown in Table 2, the prevalence of all DVTs was 18.6% in the low probability group according to PPS stratification and 27.5% in the high probability group (*p* = 0.019). Proximal DVT prevalence was higher in patients with a high PPS vs. those with a low PPS (12.7% vs. 7.1% *p* = 0.029, respectively), whereas IDDVT prevalence was similar in patients with high and low PPSs (14.7% vs. 11.6% *p* = 0.311, respectively). Considering the discriminatory accuracy of the PPS for all DVTs identified on DUS, the area under the receiver operating characteristics curve (AUC) was 0.62 ± 0.03; the AUC for proximal DVT was 0.64 ± 0.04, whereas it was 0.58 ± 0.04 for IDDVT.

**Figure 2:**
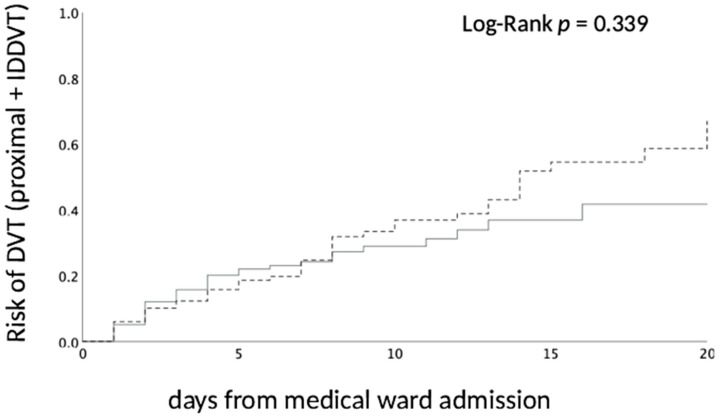
Estimated cumulative probability of all DVTs (proximal and IDDVT) in acutely ill medical inpatients suspected of DVTs with a low PPS (solid line) and in those with a high PPS (dotted line) according to the time when whole-leg ultrasonography was performed (days: day 0 from admission to the medical ward). DVT, deep vein thrombosis; IDDVT, isolated distal vein thrombosis; high PPS, Padua Prediction Score > 4; low PPS, Padua Prediction Score < 4.

The Kaplan–Meier curves of probability for DVTs in patients with a high PPS vs. low PSS according to the time from admission are reported in Figure 2 and show that patients suspected of DVTs with a high PSS had a similar risk of DVT than those with a low PPS (*p* = 0.339).

As shown in Table 3, among the 224 patients without pharmacological thrombo-prophylaxis, proximal DVT prevalence (11.6% vs. 8.0% *p* = 0.361) and IDDVT prevalence (18.6% vs. 11.6% *p* = 0.145) were similar in patients with high and low PPSs. Among the 281 patients receiving pharmacological thrombo-prophylaxis, proximal DVT prevalence was higher in patients with a high PPS vs. those with a low PPS (13.6% vs. 6.1% *p* = 0.034), whereas IDDVT prevalence was similar in patients with a high PPS vs. those with a low PPS (11.9% vs. 11.7% *p* = 0.957). Among patients with a high PPS, the prevalence of all DVTs was similar in patients with cancer vs. those without (28.6% vs. 25.8% *p* = 0.675).

## 4. Discussion

Our data show that hospital-acquired IDDVT is a more frequent finding than proximal DVT, and its prevalence in patients with a low PPS is similar than in those with a high PPS, in contrast with the aim of the PPS itself, such as the stratification of VTE risk. Of note, the PPS cannot be used to predict and diagnose DVT.

It has already been clearly established that hospitalization is one of the major factors for the risk of VTE, and hospitalization for acute medical illness is associated with an eightfold increased risk of VTE [10]. As shown by Heit et al., the overall VTE incidence rate in a cohort of patients who resided in Olmsted County, Minnesota, was 960.5 per 10,000 person-years in hospitalized patients, while it was 100 times less in community residents (7.1 per 10,000 person-years) [11]. Recently, an Italian prospective observational study performed in medicine wards showed that 0.4% of consecutive acutely ill patients developed proximal DVT prevalence during the hospital stay [12]. In our series, the estimated prevalence of all DVTs in a hospital medical setting was 1.34%, with proximal DVT incidence being estimated at 0.56%, which is in line with what has been reported in the aforementioned Italian study. Moreover, we demonstrated that most of the DVTs in inpatients from medical departments were confined to the infra-popliteal veins of the lower limbs. In accordance with these data, past and present guidelines recommend anticoagulant thrombo-prophylaxis with low-molecular-weight heparin for acutely ill hospitalized patients at an increased risk of thrombosis [4,8]. At the time of the study design, the 9th American College of Chest Physicians’ Evidence-Based Clinical Practice Guidelines suggested the PPS for VTE risk [4]. The PPS was validated based on several studies with conflicting results [13]. Vardi et al. studied VTE risk among 1080 patients hospitalized because of sepsis; they showed that 71.2% of the patients had a positive PPS, and this was highly associated with death and may reflect a more general co-morbidity and disease severity index [14]. The ESTIMATE study, the first study to test the PPS in a multicenter setting, showed that both the Geneva Risk Score and PPS were strongly associated with the composite endpoint of symptomatic VTE or VTE-related death [15]. The PPS was compared to the Caprini RAM (risk assessment model) in a Chinese case–control study by Zhou et al.; the Caprini score showed greater sensitivity in identifying high-risk hospitalized patients (82.3% of high-risk patients according to the Caprini RAM had VTE vs. 30.1% according to the PPS), even if the VTE risk associated with the highest risk groups determined by both models was similar [16]. Also, Liu et al. found that the Caprini RAM had greater sensitivity and positive and negative predictive values than the Padua RAM, although the PPS had higher specificity [17]. In addition, the Automated Padua Prediction Score (APPS) to auto-calculate the VTE risk score using electronic health records was developed, showing no significant difference in average score and a similar ability in predicting VTE risk [18].

Among the many available RAMs, in 2018, the “TEVere Score” was developed by Vincentelli et al., based on VTE risk factors with higher statistical significance, and it showed higher specificity and sensitivity (43.3 and 87.5, respectively, with 72.1 accuracy) compared with the PPS [19]. Of note, a retrospective analysis on patients prospectively enrolled in the PREVENU trial, aimed at comparing the main RAMs (including the Caprini score, IMPROVE, and PPS), showed that none of them performed significantly better than using advanced age as a single predictor [6]. Also, Wang et al. compared the PPS with nine machine learning methods, since the PPS model is not suitable for the Chinese population because of differences in race and disease spectra; nevertheless, they showed lower sensitivities in comparison to the PPS [20]. In our series, we expected a lower prevalence of DVT in the low-risk PPS group vs. those with PPS ≥ 4, whereas IDDVT prevalence was similar in patients with a high and low PPS, and proximal DVT prevalence was only slightly higher in patients with a high PPS vs. those with a low PPS. This evidence is in line with a recent review on risk assessment models for VTE in hospitalized adult patients that found a modest ability of PPS in predicting the risk of VTE [13]. In the Prevention of Venous Thromboembolism Disease in Emergency Departments (PREVENU) study on 14,660 patients hospitalized for at least 2 days in a medical ward, PPS performance was not superior to accounting for advanced age alone in VTE risk assessment [6]. Moreover, in a multicenter retrospective cohort study including over 1 million unselected consecutive hospitalizations across the United States, the PPS demonstrated limited predictive ability with a PPS discriminatory accuracy for VTE risk of 0.59 [21], which is in line with our results (discriminatory accuracy of 0.62).

Our study shows the prevalence of hospital-acquired IDDVT is higher than the prevalence of proximal DVT, in opposition with our findings in outpatients [22]. Since the prevalence of inpatient DVTs was higher than community-acquired DVTs, such differences may be at least partially due to the elevated prevalence of isolated distal DVT. Our study suggests that patients in medical wards are at higher risk of IDDVT than outpatients. In fact, the Riete registry showed that IDDVT was more frequently associated with transient risk factors (i.e., recent travel, hospitalization, recent surgery), whereas proximal DVTs were more frequently associated with chronic states [23].

Patients receiving pharmacological thrombo-prophylaxis had a similar prevalence of DVT than those without thrombo-prophylaxis. This may be due to several reasons, as follows: (1) our participants were enrolled because of suspected DVT, and this could represent a potential selection bias; (2) we performed DUS within 24 h from clinical suspicion, and this could have led to an early diagnosis before extension to the proximal veins; (3) we only enrolled patients complaining of symptoms and could have missed asymptomatic thrombosis; (4) this study was not prospective; and (5) the use of mechanical prophylaxis in several patients could have reduced DVT prevalence in patients not receiving pharmacological thrombo-prophylaxis.

The use of pharmacological thrombo-prophylaxis was similar in patients with a high and a low PPS. This is in line with a recent meta-analysis showing that thrombo-prophylaxis prescriptions were still unsatisfactory among hospitalized, medically ill patients in several countries [24]. Despite guideline recommendations, adherence to thrombo-prophylaxis remains moderate, with almost 40% of patients at high risk according to the PPS that do not receive prophylaxis [24]. These results further support a call to action for pharmacological thrombo-prophylaxis in patients because they are at risk not only for DVT but also for PE [8]. Risk assessment models should aim to help clinicians, selecting medical inpatients who are at an increased risk of VTE and may benefit from prophylaxis. However, no risk assessment model had satisfactory performances in this setting, and which risk assessment model is optimal is still uncertain. Even though not all patients may benefit from thrombo-prophylaxis, our data support the use of thrombo-prophylaxis in all medical inpatients without contraindications or high bleeding risk, as recently suggested [25].

Several limitations of this study should be acknowledged. Firstly, we must underline that our participants were enrolled because of suspected DVT; this is a potential selection bias. No inter-observer variability was assessed for IDDVT diagnosis; we did not follow-up with patients with negative whole-leg ultrasonography examinations, but several studies have shown that anticoagulant therapy can be safely withheld after negative complete compression ultrasound without further testing [22,26], also in inpatients [27]. The prevalence of DVT may have been underestimated since we did not evaluate patients with asymptomatic DVT or with symptomatic DVT who were discharged before referral to our service. Even if we excluded patients who had symptoms of DVT before hospital admission, we could not fully exclude to have enrolled some patients with DVT acquired before hospitalization. We have no data on PE prevalence since we explored only DVT prevalence in acutely ill inpatients. This study was conducted in a single academic institution and may not be representative of the population in different types of hospitals.

## 5. Conclusions

IDDVT is a frequent finding in inpatients, and its prevalence is not related to the PPS, in contrast with the aim of the PPS itself. Our study supports the consensus that clinical judgment should be integrated with risk assessment models for VTE in medical inpatients.

## Figures and Tables

**Table 1:** Characteristics of this study population (hospitalized patients with a clinical suspicion of DVT).

Age, mean (SD), year	78.0 (13.3)
Female sex	300 (59.2)
BMI mean (SD), kg/m^2^	25.4 (4.8)
Reduced mobility	258 (50.9)
Active cancer	113 (22.3)
Previous VTE	70 (13.8)
Acute infection/rheumatologic disorder	242 (47.7)
COPD	98 (19.3)
Heart Failure	78 (15.4)
Recent Trauma	73 (14.4)
Stroke	59 (11.4)
Myocardial infarction	15 (3.0)
Hormonal treatment	13 (2.6)
Thrombophilia	7 (1.4)
ICU stay	0 (0)
Hospitalization days at the time of LEUS, mean (SD), d	5.2 (6.2)
Pharmacologic thromboprophylaxis used $	282 (55.6)

Data are presented as number (percentage) of patients unless otherwise indicated. DVT, lower extremity deep vein thrombosis; LEUS, lower extremity ultrasonography study; BMI, body mass index; VTE, venous thromboembolism; COPD, chronic obstructive pulmonary disease; ICU, intensive care unit. $, use of subcutaneous heparin calcium, 5000 U, 2 or 3 times daily, or subcutaneous enoxaparin sodium, 40 mg/day, or subcutaneous fondaparinux, 1.5/2.5 mg/day.

**Table 2:** Prevalence of deep vein thrombosis in patients with suspected DVTs from medical wards according to the Padua Prediction Score.

PPS	Low Risk VTE (<4)	High Risk VTE (≥4)	*p* vs. Low Risk VTE	Total
*n* (%)	301 (59.2)	204 (40.2)		505 (100)
DVT, *n* (%)	56 (18.6)	56 (27.5)	0.019	112 (22.2)
proximal DVT, *n* (%)	21 (7.0)	26 (12.7)	0.029	47 (9.3)
IDDVT, *n* (%)	35 (11.6)	30 (14.7)	0.311	65 (12.9)
Prophylaxix, *n* (%)	163 (54.2)	118 (57.8)	0.413	281 (55.6)

Chi-square test *p*-value. VTE, venous thromboembolism; DVT, lower extremity deep vein thrombosis; IDDVT, isolated distal or calf DVT; PPS, Padua Prediction Score; Prophylaxix, pharmacologic thromboprophylaxis defined as the use of subcutaneous heparin calcium, 5000 U, 2 or 3 times daily, or subcutaneous enoxaparin sodium, 40 mg/day, or subcutaneous fondaparinux, 1.5/2.5 mg/day.

**Table 3:** Number of acutely ill medical inpatients suspected of DVTs with lower extremity vein thrombosis at DUS according to the presence or absence of pharmacological prophylaxis.

PPS	<4 (Low Risk VTE)		≥4 (High Risk VTE)	
	Prophylaxis −	Prophylaxis +	Prophylaxis −	Prophylaxis +
DUS Negative	11 (80.4)	134 (82.2)	60 (69.8)	88 (74.6)
IDDVT	16 (11.6)	19 (11.7)	16 (18.6)	14 (11.9)
DVT	11 (8.0)	10 (6.1)	10 (11.6)	16 (13.6)
Total DVT	27 (19.6)	29 (17.8)	26 (30.2)	30 (25.5)

Data are presented as number (percentage). DUS, whole-leg duplex ultrasound; DVT, proximal deep vein thrombosis; IDDVT, isolated distal deep vein thrombosis. PPS, Padua Prediction Score; Prophylaxis +, use of subcutaneous heparin calcium, 5000 IU, 2 or 3 times daily, or subcutaneous enoxaparin sodium, 40 mg/day, or subcutaneous fondaparinux, 1.5/2.5 mg/day, Prophylaxis −, patients without pharmacological thrombo-prophylaxis.

## Data Availability

The raw data supporting the conclusions of this article will be made available by the authors on request.
